# Erschwerte Diagnosestellung eines Osteoidosteoms der Großzehe: Fallbericht mit Review der Literatur

**DOI:** 10.1007/s00132-021-04082-z

**Published:** 2021-04-08

**Authors:** Ruth Thiemann, Hans-Werner Seide, Klaus-Dieter Luitjens, Frank Timo Beil, Tim Rolvien, Lara Krüger

**Affiliations:** 1 Klinik für Unfallchirurgie und Orthopädie, Westküstenkliniken Brunsbüttel und Heide gGmbH, Brunsbüttel, Deutschland; 2 Orthopädische Universitätsklinik, Klinikum Bad Bramstedt GmbH, Bad Bramstedt, Deutschland; 3 Fachbereich Orthopädie, Klinik für Unfallchirurgie und Orthopädie, Universitätsklinikum Hamburg-Eppendorf (UKE), Martinistraße 52, 20246 Hamburg, Deutschland

**Keywords:** Fußknochen, Hallux, Makrodaktylie, Neoplasie, Schmerzen, Foot bones, Hallux, Macrodactyly (local gigantism), Neoplasia, Pain

## Abstract

**Hintergrund:**

Osteoidosteome zählen zu den benignen ossären Neoplasien und können am gesamten Skelett auftreten. Die verlängerte Diagnosestellung eines Osteoidosteoms der Großzehe veranlasste uns, einen Fallbericht mit Review der Literatur zu verfassen, um mögliche Fehlerquellen zu identifizieren und einen Beitrag zur zügigeren Diagnosefindung und Therapie zu leisten.

**Literatur:**

In der deutschen Literatur sind aktuell nur zwei Fallberichte zu Osteoidosteomen der Zehen bekannt. International wurde allerdings eine Vielzahl an Fällen beschrieben, die in der Zusammenschau ein homogenes Bild ergeben. Eine sehr deutliche Häufung von Osteoidosteomen am Endglied der Großzehe lassen eine posttraumatische Ätiologie möglich erscheinen. Der typische NSAR-sensible Nachtschmerz ist klinisch eindeutig und sollte an jeder Lokalität des Körpers an ein Osteoidosteom denken lassen. Auffällig ist das an den Zehen einzigartige Symptom der Makrodaktylie, was ein dankbarer Hinweis zur Diagnosefindung sein kann. Die mittlere Zeit vom Symptombeginn bis zur korrekten Diagnosestellung betrug 12 Monate. Häufig wurden Fehldiagnosen und Fehltherapien unter einer anderen Verdachtsdiagnose beschrieben. Eine mögliche Ursache ist die häufig atypische und uneindeutige Bildgebung. Die operative Therapie stellt bei Osteoidosteomen der Zehen die Methode der Wahl dar und sollte stets durch eine histopathologische Untersuchung ergänzt werden.

## Abstract (en)

**Background:**

Osteoid osteomas belong to the benign osseous neoplasia and can occur on the entire skeleton. The prolonged diagnosis of an osteoid osteoma of the great toe prompted us to prepare a case report with a literature review to identify possible sources of error and to contribute to a more expeditious diagnosis and therapy.

**Literature:**

Currently, only two case reports on osteoid osteomas of the toes are known in the German literature. Internationally, however, a large number of cases have been described, which, when viewed as a whole, present a homogeneous picture. A very clear accumulation of osteoid osteomas at the distal phalanx of the great toe suggests a possible post-traumatic aetiology. The typical NSAID-sensitive night pain is clinically unambiguous and should make one think of an osteoid osteoma at any site on the body. The symptom of macrodactyly, which is unique to the toes, is striking and may provide a helpful clue to establishing the diagnosis. The median time from symptom onset to correct diagnosis was 12 months. One possible reason for this prolonged diagnosis is the often atypical and ambiguous imaging. Surgical therapy is the method of choice for osteoid osteomas of the toes and should always be supplemented by histopathologic examination.

Osteoidosteome sind benigne ossäre Neoplasien, sie können überall am Skelett auftreten. An den Zehen gehören sie allerdings zu den Raritäten, auch wenn international einige Fälle beschrieben sind. Aufgrund der Seltenheit an den Zehen ist die korrekte Diagnosestellung häufig stark verzögert. Die operative Therapie ist die Therapie der Wahl und die Diagnose sollte immer mittels histopathologischer Untersuchung gesichert werden.

## Falldarstellung

### Anamnese

Eine 37-jährige Patientin ohne relevante Vorerkrankungen klagte seit 12 Monaten über druckabhängige Schmerzen des Endglieds der rechten Großzehe, betont beim Tragen von engem Schuhwerk. Es entwickelte sich ein Belastungsschmerz sowie im Verlauf auch Dauerschmerz bis NRS (Numerische Rating-Skala) 7/10 mit deutlicher Beschwerdezunahme abends und nachts. Nach Einnahme von NSAR (nichtsteroidale Antirheumatika), Metamizol oder Paracetamol kam es zu einer unmittelbaren Beschwerdebesserung, es zeigte sich keine Überlegenheit eines Analgetikums. Anamnestisch konnte die Patientin kein auslösendes Ereignis oder kürzliches Trauma angeben. Als passionierte Reiterin berichtete sie lediglich über rezidivierende Quetschtraumata durch Tritte des Pferdes auf den Fuß vor vielen Jahren.

### Klinischer Befund

**Figure 1:**
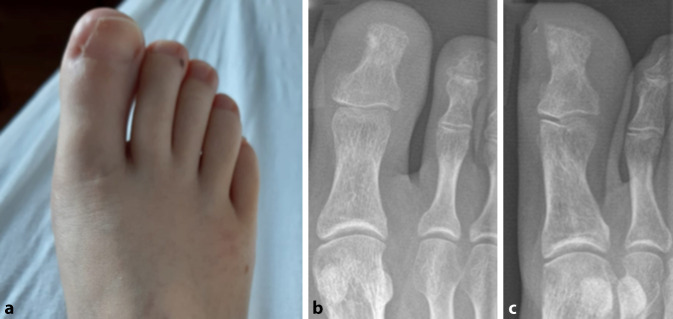

Die rechte Großzehe zeigte sich im Seitenvergleich dezent gerötet, überwärmt und geschwollen ohne Anhalt von Pus (Abb. 1). Die Beweglichkeit im Grundgelenk und dem Interphalangealgelenk war schmerzbedingt gehemmt, passiv aber nicht eingeschränkt. In der Palpation klagte die Patientin über eine diffuse Berührungsempfindlichkeit.

### Diagnostik

Bei der Verdachtsdiagnose Unguis incarnatus mit Differenzialdiagnose Osteomyelitis erfolgte extern die Röntgendiagnostik der Großzehe. Hier zeigte sich eine geringe Sklerosierung am medialen Endglied (Abb. 1) und kein Nachweis von Osteolysen. Die Laborwerte waren stets unauffällig. Es erfolgte eine Nagelkeilexzision, die keine suffiziente Beschwerdelinderung erbrachte. Bei ausbleibender Besserung und dezenter Sekretion am medialen Nagelrand erfolgte 2 Monate postoperativ mit der Verdachtsdiagnose eines Rezidivs eine erneute Nagelkeilexzision mit sparsamem knöchernem Débridement. Es erfolgte keine histologische Untersuchung. Differenzialdiagnostisch kam ein infektiöser Prozess weiterhin in Betracht, weswegen eine probatorische antibiotische Therapie mit Cefuroxim erfolgte. Auch hierunter zeigte sich keine Beschwerdelinderung.

**Figure 2:**
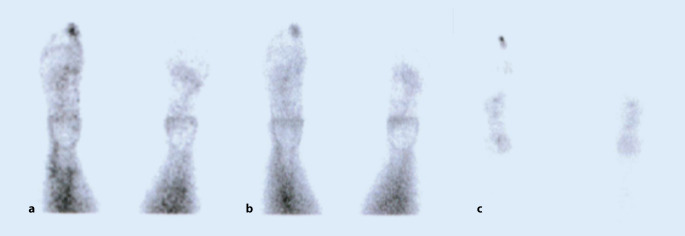

Zehn Monate nach Beschwerdebeginn erfolgte eine 3‑Phasen-Szintigraphie. Differenzialdiagnostisch wurde hier bereits ein Osteoidosteom in Erwägung gezogen. Die Szintigraphie zeigte eine deutliche Hyperperfusion in der arteriellen Phase am Großzehenendglied (Abb. 2). Aufgrund der diffusen Perfusion des Endglieds, die nicht nur den Nidus betraf, wurde im radiologischen Befund ein Osteoidosteom als nicht wahrscheinlich beschrieben. Am ehesten sei der Befund passend zum Bild eines Glomustumors.

**Figure 3:**
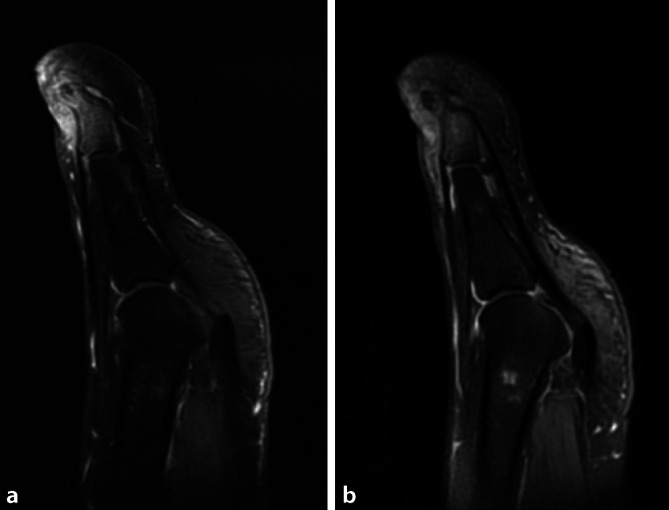

Eine MRT (Magnetresonanztomographie) zeigte in der fettsupprimierten T2-Wichtung eine hyperintense Signalalteration des Endglieds im Sinne eines Knochenmarködems sowie der angrenzenden Weichteile mit hypointenser Darstellung in der T1-Wichtung. In der Kontrastmittelphase zeigte sich eine vermehrte Kontrastmittelaufnahme der Weichteile und des knöchernen Endglieds mit hypointenser Demarkation medial am Endglied (Abb. 3). Im radiologischen Befund wurde dies als hochgradiger Verdacht auf eine Osteomyelitis bei Vorhandensein einer Kompaktainsel des medialen Endglieds gewertet, ebenso nicht passend zur Verdachtsdiagnose eines Osteoidosteoms.

**Figure 4:**
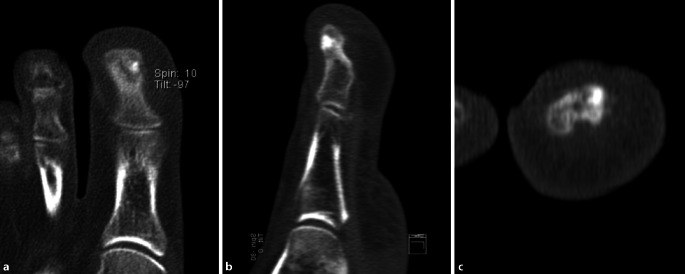

Eine interventionelle Probenentnahme war aufgrund der geringen Größe der Läsion von 7 × 4 mm und der direkt subungualen Lage nicht möglich. Die Patientin beschrieb einen hohen Leidensdruck und klagte fortwährend über eine deutlich eingeschränkte Lebensqualität mit dauerhafter Analgetikaeinnahme, eingeschränkter Gehstrecke und persistierenden Schmerzen. Ein Jahr nach Beschwerdebeginn erfolgte aufgrund der spezifischen Anamnese letztendlich eine Dünnschicht-CT (Abb. 4). Diese zeigte eine ovale Aufhellung mit zentraler punktueller Verschattung und umgebender Sklerosierung (im Sinne eines klassischen Nidus) und letztendlich den hochgradigen Verdacht auf ein Osteoidosteom.

Aufgrund der subungualen Lage schien wegen drohender Kollateralschäden eine Radiofrequenzablation wenig geeignet. Daraufhin erfolgte die Vorstellung in unserer orthopädischen Universitätsklinik und die Indikationsstellung zur operativen Resektion.

### Therapie und Verlauf

Es erfolgte 14 Monaten nach Beschwerdebeginn die knöchern Partialresektion des distalen Endglieds über einen fischmaulartigen Zugang ca. 4 mm unterhalb des Zehennagels. Auch äußerlich sah man eine Verdickung medialseitig am betroffenen knöchernen Bereich. Mittels oszillierender feiner Säge erfolgte die nahezu hälftige Resektion des Endglieds.

**Figure 5:**
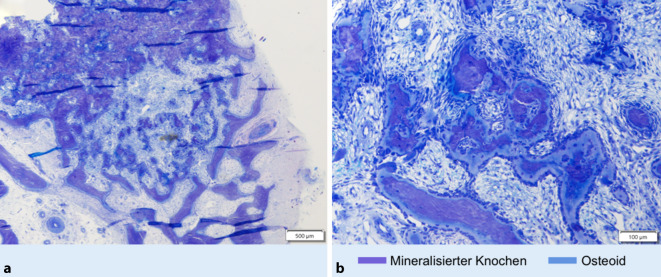

Histologisch bestätigte sich die Verdachtsdiagnose des Osteoidosteoms. In der unentkalkten Aufarbeitung des Resektats wurde der Nidus, bestehend aus nichtmineralisierter Knochenmatrix (Osteoid) und zentraler Sklerose, vollständig abgebildet (Abb. 5). Die orale Analgesie konnte nach einer Woche vollständig abgesetzt werden. Zuvor bestehende nächtliche Schmerzen waren unmittelbar vollständig regredient. Die Mobilisation erfolgte postoperativ für 4 Wochen im Verbandsschuh, zu Beginn an Unterarmgehstützen.

Die Wundheilung erfolgte primär, der Fadenzug zeitgerecht. Nach 3 Monaten war kein Unterschied mehr im Gangbild zu erkennen, im 1‑Jahres-Follow-Up bestand lediglich noch eine residuelle Berührungsempfindlichkeit. Die Patientin ist im Alltag und bei moderater Belastung nicht eingeschränkt.

## Literaturreview

Am 28.11.2020 erfolgte eine systematische Recherche der englischen und deutschen Literatur in Medline und Google Scholar mit den Suchbegriffen „osteoid osteoma“, „toe“, „hallux“, „phalanx“ und „foot“. Eingeschlossen wurden sämtliche Fallberichte über Osteoidosteome an den Zehen sowie Reviews, die Fallberichte enthielten. Ausgeschlossen wurden Artikel, die aufgrund einer fehlenden doi- oder PMID-Nummer nicht über die universitäre Fernleihe erhältlich waren oder für die kein Abstract zur Verfügung stand. Nach Ausschluss von Duplikaten konnten insgesamt 231 Artikel identifiziert werden. Anhand der Titel konnten weitere 111 Artikel ausgeschlossen werden. Durch Beurteilung der Abstracts wurden weitere 76 Artikel ausgeschlossen. Nach Volltextsichtung der verbliebenen 44 Artikel konnten letztendlich 34 Fachartikel mit insgesamt 37 berichteten Fällen im Zeitraum von 1975 bis 2020 eingeschlossen werden (Tab. 1; [2, 3, 5–9, 11–13, 15, 16, 18–27, 30, 32–37, 39–43]).

**Table 1:** 

Autor und Jahr	Alter	m/f	Zeh	Glied	Symptome	NSAR-sensibel	Monate bis Diagnose	Bildgebung	Differenzialdiagnosen	Therapie	Follow-Up-Zeit Rezidiv?
Adler et al. 1997 [2]	23	m	1	EG	Nachtschmerz Schwellung Überwärmung Hypersensibilität	Vollständig	23	Röntgen Szintigraphie MRT Histo definitiv	Entzündlich (Osteomyelitis) rheumatoide Grunderkrankung	OP	KA
Alkalay et al. 1987 [3]	22	m	1	GG	Nachtschmerz Schwellung	Vollständig	12	Röntgen Histo definitiv	Trauma (Z. n. Sturz)	OP	1 Jahr
Baller et al. 2000 [5]	27	f	2	EG	Schmerz (nicht tageszeitenabhängig) Makrodaktylie	Vollständig	9	Röntgen Histo definitiv	Entzündlich (Osteomyelitis) anderer Tumor	OP	KA
Barca et al. 1998 [6] *1. Fall*	24	m	2	EG	Nachtschmerz Makrodaktylie Überwärmung Rötung	KA	30	Röntgen Szintigraphie Histo definitiv	Trauma	OP	3 Jahre
*2. Fall*	30	m	3	EG	Nachtschmerz Überwärmung Makrodaktylie	KA	24	Röntgen Histo definitiv	KA	OP	5 Jahre
Başar et al. 2014 [7]	34	m	1	EG	Nachtschmerz Schwellung	Vollständig	17	Röntgen CT MRT Histo definitiv	Entzündlich	OP	36 Monate
Basile et al. 2020 [8]	27	f	1	GG	Nachtschmerz keine weiteren Symptome	Teilweise	13	Röntgen CT MRT Szintigraphie Histo definitiv	KA	OP	12 Monate
Bordelon et al. 1975 [9]	14	m	1	EG	Nachtschmerz Makrodaktylie Druckschmerz keine Rötung	Vollständig	12	Röntgen Histo definitiv	RA	OP	KA
Cinka et al. 2019 [11]	22	m	2	EG	Schmerz (KA wann) Makrodaktylie keine Rötung	Vollständig	4	Röntgen CT Histo definitiv	Entzündlich (Osteomyelitis) anderer Tumor (Enchondrom, Glomustumor) Epidermoidzyste	OP	9 Monate
Ebrahimzadeh et al. 2009 [12]	20	m	2	EG	Nachtschmerz Rötung Makrodaktylie Überwärmung	Vollständig	6	Röntgen Szintigraphie Histo definitiv	KA	OP	1 Jahr
Ekmekci et al. 2001 [13]	29	f	1	EG	Knotiger Tumor subungual schmerzfrei	Kein Schmerz	KA	Röntgen Histo definitiv	Anderer Tumor (Exostose, Osteochondrom, Osteom)	OP	8 Monate
Hamilos und Cervetti 1987 [15]	37	f	1	EG	Nachtschmerz Schwellung Rötung	Vollständig	KA	Röntgen Histo definitiv	Bandscheibenvorfall Gicht Psychosomatisch	OP	6 Jahre
Hattori et al. 2011 [16]	22	m	1	EG	Nachtschmerz leichte Schwellung	Teilweise	8	Röntgen CT MRT Histo definitiv	Entzündlich (Osteomyelitis, Abszess) anderer Tumor (Glomustumor, Epidermoidzyste)	OP	8 Monate
Jowett und Singh 2010 [18]	20	f	1	EG	Nachtschmerz Schwellung IP-Beweglichkeit eingeschränkt leichte Temperaturerhöhung	Teilweise	36	Röntgen CT Histo definitiv	KA	OP	KA
Kahn et al. 1983 [19]	32	f	1	EG	Nachtschmerz Rötung	KA	12	Röntgen Szintigraphie CT Histo definitiv	Entzündlich (Paronychie, Osteomyelitis, Abszess) anderer Tumor (Enchondrom, maligne, Knochenzyste, Glomustumor)	OP	7 Monate
LaCroix et al. 2001 [20]	7	m	4	EG	Schmerz (KA wann) Schwellung	KA	KA	KA Histo definitiv	Entzündlich anderer Tumor (Osteoblastom)	OP	2 Monate
Lakkis et al. 1998 [21]	32	m	1	GG	Schmerz (KA wann) Schwellung	Keine Besserung	24	Röntgen MRT Histo definitiv	Degenerativ	OP	12 Monate
Meng und Watt 1989 [22] *3 Fälle*	KA	KA	1 5	3 × EG	3 × Schmerz (KA wann) 3 × Schwellung	KA	1 × 7	Röntgen	3 × Fehldiagnose zuvor	3 × OP	KA
Mohr et al. 1990 [23]	20	m	1	EG	Schmerz (KA wann) Makrodaktylie Verfärbung Nagelveränderungen	KA	21	Röntgen Kürettage mit PE Histo definitiv	Entzündlich (Osteomyelitis)	OP	4 Monate
Mohsen et al. 2015 [24]	32	f	1	EG	Schmerz (KA wann) Schwellung	Vollständig	8	Röntgen MRT Histo definitiv	Rheumatoide Grunderkrankung	OP	KA
Onoue und Kudawara 2007 [25]	30	m	4	EG	Nachtschmerz Makrodaktylie Rötung	Teilweise	5	Röntgen MRT Szintigraphie Histo definitiv	Entzündlich (Osteomyelitis)	OP	2 Jahre
Oztürk et al. 2008 [26]	9	f	1	EG	Schmerz (KA wann) Makrodaktylie	KA	24	Röntgen CT Histo definitiv	KA	OP	12 Monate
Prietzel et al. 2009 [27]	12	f	2	EG	Schmerz (tageszeitenunabhängig) Makrodaktylie	KA	12	Röntgen MRT Szintigraphie Histo definitiv	Trauma (Z. n. Pferdetritt) entzündlich (Osteomyelitis) anderer Tumor rheumatoide Grunderkrankung	OP	18 Monate
Shader und Schwartzenfeld 1989 [30]	38	f	5	GG	Schmerz (KA wann) Makrodaktylie	KA	108	Röntgen Histo definitiv	Anderer Tumor (Osteom, Osteoblastom, Osteosarkom)	OP	24 Monate
Spinosa et al. 1985 [32]	29	f	1	EG	Nachtschmerz keine weiteren Symptome	Paracetamol teilweise (ASS-Allergie)	12	Röntgen Histo definitiv	Entzündlich anderer Tumor (Exostose)	OP	9 Monate
Sproule et al. 2004 [33]	14	m	2	EG	Nachtschmerz Makrodaktylie Rötung Überwärmung	Vollständig	8	Röntgen Szintigraphie Histo definitiv	Entzündlich anderer Tumor (Osteoblastom, Glomustumor) Epidermoidzyste	OP	12 Monate
Torrent et al. 2017 [34]	16	m	1	GG	Schmerz (KA wann) IP-Beweglichkeit eingeschränkt	KA	KA	Röntgen CT Histo definitiv	Rheumatoide Grunderkrankung anderer Tumor (Enchondrom, Riesenzelltumor, aneurysmatische Knochenzyste)	OP	6 Monate später Rezidiv Follow Up: 30 Monate
Trave et al. 2020 [35]	KA	f	1	EG	Schmerz (KA wann) Makrodaktylie Nagelveränderung keine Rötung oder Überwärmung	Vollständig	12	Röntgen	Entzündlich (Onychomykose)	Konservativ (ASS)	KA
Tsang und Wu 2008 [36]	38	m	4	EG	Nachtschmerz Schwellung Nagelveränderung	Vollständig	24	Röntgen Histo definitiv	KA	OP	11 Jahre
Turkmen et al. 2013 [37]	23	m	1	EG	Nachtschmerz Makrodaktylie Überwärmung	Vollständig	24	Röntgen MRT Szintigraphie Histo definitiv	Entzündlich (Osteomyelitis)	OP	10 Monate
Wang et al. 2019 [39]	12	m	2	EG	Schmerz (KA wann) Makrodaktylie Hyperhidrose	KA	10	Röntgen	Anderer Tumor	OP	24 Monate
Woo et al. 2019 [40]	53	m	1	EG	Nachtschmerz Makrodaktylie Nagelvergrößerung	Teilweise	18	MRT	Entzündlich (Paronychie, Onychomykose) anderer Tumor (Glomustumor, Sarkom) Neurofibromatose, Tuberous sklerosis, Klippel-Trenaunay-Syndrom, Trisomie 21 Proteus-Syndrom	OP	KA
Wu 1991 [41]	17	f	3	EG	Schmerz (KA wann) Makrodaktylie	Teilweise	13	Röntgen Szintigraphie Histo definitiv	KA	OP	KA
Xarchas et al. 2017 [42]	22	m	1	EG	Nachtschmerz Makrodaktylie keine Rötung oder Überwärmung	Teilweise	18	Röntgen CT Histo definitiv	Entzündlich (Osteomyelitis) anderer Tumor	OP	2 Jahre
Yamaga et al. 2015 [43]	16	m	1	EG	Schmerz (KA wann) Makrodaktylie Rötung	KA	10	Röntgen CT MRT Histo definitiv	Entzündlich (Osteomyelitis)	OP	KA

*m* männlich; *f* weiblich; *EG* Endglied; *GG* Grundglied; *NSAR* nichtsteroidale Antirheumatika; *MRT* Magnetresonanztomographie; *CT* Computertomographie; *OP* Operation; *KA* Keine Angabe; *ASS* Acetylsalicylsäure; *IP* Interphalangealgelenk

Das mittlere Alter dieser Patienten betrug 24,3 Jahre (7–53 Jahre). Das Verhältnis männlich zu weiblich betrug 1,6:1. Am häufigsten war die Großzehe betroffen (*n* = 22, 61 %), die übrigen Zehen waren seltener betroffen (2. Zehe: *n* = 7, 19 %; 3. Zehe: *n* = 2, 6 %; 4. Zehe: *n* = 3, 8 %; 5. Zehe: *n* = 2, 6 %). In der überwiegenden Anzahl der Fälle war das Endglied betroffen (*n* = 31, 86 %).

Bei 36 der 37 Patienten (97 %) berichteten die Autoren von Schmerzen, in 19 Fällen (53 %) wurde eine nächtliche Schmerzverstärkung beschrieben. Lediglich ein Patient (3 %) wurde als schmerzfrei beschrieben. Von einer Makrodaktylie bzw. Schwellung der Zehe wurde bei 32 Patienten (86 %) berichtet. Eine Rötung und/oder Überwärmung der betroffenen Zehe wurde in 11 Fällen (30 %) beschrieben.

Bei 22 der 36 schmerzgeplagten Patienten lagen Angaben zur Analgetikatherapie vor. Davon berichteten 13 Patienten (59 %) von einem sehr guten bis guten Ansprechen auf NSAR und 8 Patienten (36 %) von einem geringen Ansprechen auf NSAR. Ein Patient (5 %) beschrieb keine Schmerzbesserung.

Als bildgebende Diagnostik erhielten 35 Patienten ein Röntgenbild, 11 Patienten eine MRT, 10 Patienten eine CT und 10 Patienten eine Szintigraphie. Ob die durchgeführten bildgebenden diagnostischen Maßnahmen vollständig angegeben wurden, kann nicht beurteilt werden. Ab Symptombeginn vergingen im Median 12 Monate (4–108 Monate) bis zur korrekten Diagnosestellung.

Als häufigste Differenzialdiagnosen wurden eine Infektion (*n* = 17, in der Regel V. a. Osteomyelitis oder Paronychie) oder ein benigner oder maligner Tumor (*n* = 14) in Betracht gezogen. Bei 7 Patienten (19 %) waren eine oder mehrere vorhergehende Operationen unter einer anderen Verdachtsdiagnose beschrieben [16, 19–21, 23, 27, 32]. 36 der 37 Patienten (97 %) wurden operativ, zumeist mittels En-bloc-Resektion, therapiert. In 100 % der operativ therapierten Patienten erbrachte die anschließende histopathologische Befundung ein eindeutiges Ergebnis.

Die Follow-Up-Dauer betrug im Median 12 Monate (2–132 Monate). Bei einem Patienten wurde bei unvollständiger Resektion des Nidus ein Rezidiv beschrieben, welches eine erneute operative Therapie erforderte.

## Diskussion

Trotz der typischen Anamnese und klinischen Symptome von Osteoidosteomen sind sie an den Zehen mit einer verlängerten Diagnosestellung und mit häufigen Fehldiagnosen und -therapien assoziiert. Ziel dieses Fallberichts und Literaturreviews war es, durch die Betrachtung unseres Falles sowie aller publizierten Fälle mögliche Fehlerquellen zu identifizieren, um einen Beitrag zur zügigeren Diagnosefindung und Therapie zu leisten.

Osteoidosteome zählen zu den benignen ossären Neoplasien, von denen sie ca. 10 % ausmachen [14]. Histopathologisch findet sich ein zentraler, osteoidreicher Nidus mit einem hohen Anteil an Osteoblasten und einer umgebenden reaktiven Sklerosezone, vermutlich als Zeichen einer physiologischen Reaktion des umgebenden Knochens auf den erhöhten intraossären Druck [14]. Immunhistochemisch wurden Prostaglandine (PGE_2_, PGI_2_, PGF_2_α) in hoher Konzentration im Nidus nachgewiesen. Deren vasodilatativen und angiogenetischen Effekte scheinen die intraossäre Drucksteigerung und ein entsprechendes perifokales, schmerzhaftes Knochenödem zu bedingen [14, 28].

Prinzipiell können Osteoidosteome am gesamten Skelett auftreten. Mit über 50 % der Fälle sind allerdings die häufigste Prädilektionsstellen die langen Röhrenknochen des Femurs oder der Tibia [14, 29]. Der Fuß und das Sprunggelenk sind mit 2–10 % der Fälle eine seltene Lokalität, hier ist vor allem der Talus betroffen [14, 17]. Zu Osteoidosteomen der Zehen sind aus der deutschen Literatur aktuell nur zwei Fallberichte bekannt [23, 27].

Im Review der internationalen Literatur fällt allerdings eine Vielzahl an Fällen auf, die in der Zusammenschau ein homogenes Bild ergeben. Das Überwiegen des männlichen Geschlechts sowie ein vermehrtes Auftreten in der 2. und 3. Lebensdekade decken sich mit der bekannten üblichen Verteilung am übrigen Körper [14]. Im Literaturreview zeigt sich eine sehr deutliche Häufung von Befunden am Endglied der ersten Zehe. Diese war auch im eigenen beschriebenen Fall betroffen. Ungewöhnlich ist das Auftreten bei einer weiblichen, bereits 37-jährigen Patientin.

Die Ätiologie und Pathogenese des Osteoidosteoms sind aktuell noch nicht vollständig verstanden [14]. Die Entstehung aus einem atypischen Heilungsprozess nach einer Entzündungsreaktion oder einem Trauma wird diskutiert [38]. Der Zusammenhang mit dem erhöhten Knochenstoffwechsel der 2. und 3. Lebensdekade wird vermutet [14]. In Anbetracht der exponierten Lage des Zehenendglieds erscheint ein Entstehen im Rahmen von posttraumatischen Regenerationsprozessen durchaus möglich. Ein Trauma in der Anamnese wurde in bis zu einem Drittel der Fälle beschrieben [14]. Gurkan und Kollegen beschrieben eine histologische Ähnlichkeit der Nidusentstehung und der desmalen Ossifikation, die im Rahmen einer Frakturheilung auftritt [14]. Adil und Kollegen vermuteten eine traumatisch aufgetretene Invagination des Periosts als möglichen Prädispositionsfaktor für die Entstehen eines Osteoidosteoms [1]. Folglich könnte der Nidus ein versprengter atopischer Ossifikationsbereich im Knochen sein. Die Ursache der ausbleibenden Reifung des Nidus ist nicht abschließend geklärt [14].

Das typische Symptom „NSAR-sensibler Nachtschmerz“ ist klinisch sehr eindeutig [17] und sollte an jeder Lokalität des Körpers an ein Osteoidosteom denken lassen. Im Review der Literatur fällt aber das für diese Körperstelle einzigartige Symptom der Makrodaktylie, teils mit Nagelveränderungen, auf. Dies bietet einen dankbaren Hinweis zur klinischen Diagnosefindung. Trotz dieser teils deutlichen klinischen Hinweise scheint die Diagnose des Osteoidosteoms an den Zehen eine Herausforderung zu sein. Vom initialen Symptom bis zur korrekten Diagnosestellung vergingen im Median 12 Monate. Andere Autoren beschrieben am gesamten Fuß einen Zeitraum von im Mittel 18, bzw. 22 Monaten [14, 17]. Die häufigste beschrieben Fehldiagnose war die Infektion, in der Regel im Sinne einer Osteomyelitis oder Paronychie. Durch die in vielen Fällen auftretende Rötung und Überwärmung liegt die Verdachtsdiagnose einer Infektion nahe. Diese Symptome können durch den geringen Hautmantel über der Läsion und auftretende vasomotorische Störungen bedingt sein [41]. Durch ein gestörtes Nagelwachstum kann weiterhin eine Paronychie entstehen, welche die ursprünglich eindeutigen klinischen Symptome verschleiert.

Eine weitere Besonderheit des Osteoidosteoms an den Zehen stellt die häufig atypische und uneindeutige Bildgebung dar [22, 31], die auch die Diagnosestellung im eigenen beschriebenen Fall erschwerte. Nativradiologisch wurde ein häufiges Fehlen der ansonsten sehr typischen Sklerosezone beschrieben [17, 22, 31]. Eine Erklärung ist die häufig intramedulläre oder subperiostale Lage des Osteoidosteoms an den Füßen, die in der Regel keine starke periostale Reaktion zeigen [6, 17]. Bei intramedullären Osteoidosteomen wird weiterhin eine mögliche knöcherne Expansion beschrieben [22]. Die subperiostale Lage geht häufig mit einer juxtakortikalen Weichteilverdickung einher [22]. Die geringe periostale Reaktion mit ausbleibender Sklerosebildung wurde ebenfalls an den Händen beschrieben [22]. Meng und Kollegen vermuteten ursächlich die im Verhältnis zum Schaft sehr dünne Kortikalis an den feinen Knochen von Hand und Fuß sowie wenig vorhandenes Periost durch die engmaschigen Sehnenansätze dieser anatomischen Regionen [22]. Die geläufigeren Osteoidosteome der langen Röhrenknochen sind hingegen häufig intrakortikal lokalisiert [17] und stellen sich mit der klassischen dichten umgebenden Sklerosezone dar.

In der Szintigraphie ist die hohe Aktivität der Läsion nachweisbar, welche kaum eine Differenzierung zu anderen aktiven Neoplasien oder einem Infektgeschehen zulässt [22]. Bei sehr feiner Anatomie der Zehen ist die Auflösung zur Darstellung des ansonsten häufig beschriebenen Double-Density-Sign nicht ausreichend. MRT-morphologisch ist häufig ein sehr ausgeprägtes Knochenmarksödem ersichtlich, der Nidus kann am Fuß nur in 67 % der Fälle dargestellt werden [28, 31]. Von anderen Autoren wurde eine Fehlerquote in der MRT-Diagnostik von 33–34 % beschrieben [14, 17]. Vorsicht ist weiterhin bei der Interpretation des radiologischen Befunds geboten, der in der Regel ohne Kenntnis der Anamnese inklusive vorheriger Operationen erstellt wurde.

Lediglich die Dünnschicht-CT bietet eine nahezu pathognomonische Darstellung [31] und ist der MRT in der Diagnose des Osteoidosteoms überlegen [4, 31]. Andere Autoren beschrieben eine Diagnosesicherheit von 96–100 % [14, 17].

An den Zehen stellt die operative Therapie in der Regel die Behandlung der Wahl dar. Dies ist eine Besonderheit, da an den langen Röhrenknochen die Radiofrequenzablation mittlerweile als Goldstandard der Therapie angesehen werden kann [10]. Diese ist wenig invasiv und hat sich als sicher und hoch effektiv erwiesen. Es werden Erfolgsraten von 76–100 % beschrieben [10]. An den Zehen kommt diese häufig nicht infrage. Ursächlich ist die häufig unklare Dignität der Läsion, die sehr kleine Läsionsgröße, zu erwartende Hautnekrosen durch die Hitze der Nadelspitze und die unmittelbare Nähe zu neurovaskulären Strukturen an den Zehen [10]. Bei vollständiger Resektion des Nidus werden auch bei der operativen Therapie Erfolgsraten von 88–100 % beschrieben [10].

Die intraoperativ entnommene Histologie bot in allen beschriebenen Fällen den definitiven Beweis und sollte bei operativen Eingriffen mit unklarer zugrundeliegender Pathologie stets erfolgen. Auch die klinisch und bildmorphologisch teils nur schwer zu unterscheidende Differenzialdiagnose der Infektion lässt sich durch die intraoperative Probenentnahme sicher beurteilen.

## Limitationen

Die im Review der Literatur dargestellten Daten wurden von anderen Autoren erhoben und publiziert und es ist unklar, ob sämtliche relevanten Informationen zur Verfügung gestellt wurden. Die Inzidenz von Osteoidosteomen der Zehen in der Bevölkerung bleibt unklar.

## Fazit für die Praxis

- Osteoidosteome an den Zehen sind mit einer verlängerten Diagnosestellung sowie häufigen Fehldiagnosen und -therapien assoziiert.
- Klinisch und bildmorphologisch zeigen sich einige Besonderheiten, die die Diagnosestellung erschweren können.
- Der NSAR(nichtsteroidale Antirheumatika)-sensible Nachtschmerz kann an sämtlichen Stellen des Körpers auf ein Osteoidosteom hinweisen.
- Die Makrodaktylie einer Zehe stellt eine besondere klinische Ausprägung dar.
- Am häufigsten ist das Endglied der Großzehe betroffen. Eine mögliche posttraumatische Ätiologie kann diskutiert werden.
- Die klassische umgebende Sklerosierung im Röntgenbild kann an den Zehen fehlen.
- Eine Szintigraphie- und MRT-Darstellung bieten nur eine begrenzte diagnostische Aussagekraft und häufig keine ausreichende Abgrenzung zur Differenzialdiagnose „Infektion“.
- Die Dünnschicht-CT bietet die höchste diagnostische Sicherheit und sollte stets in der diagnostischen Schleife enthalten sein.
- Die operative Therapie ist in der Regel die Methode der Wahl.
- Die intraoperative Probenentnahme sollte stets bei allen Eingriffen mit unklarer zugrunde liegender Pathologie erfolgen. In Anbetracht der klinischen und bildmorphologischen Besonderheiten bietet nur die Histologie die definitive Bestätigung der Verdachtsdiagnose.

## Abkürzungen

ASS: Acetylsalicylsäure
EG: Endglied
GG: Grundglied
IP: Interphalangealgelenk
NRS: Numerische Rating-Skala
NSAR: Nichtsteroidale Antirheumatika
PG: Prostaglandin
